# *Nicotinamide phosphoribosyltransferase* (Nampt) in Lateral Hypothalamus Maintains Skeletal Muscle Functions Through Lactate‐Mediated Calcium Signalling in Male Mice

**DOI:** 10.1002/jcsm.70055

**Published:** 2025-09-16

**Authors:** Takahiro Eguchi, Keiko Kabetani, Naoki Ito

**Affiliations:** ^1^ Brain‐Skeletal Muscle Connection in Aging Project Team, Geroscience Research Center National Center for Geriatrics and Gerontology Japan

**Keywords:** Ca^2+^ signalling, lactate, lateral hypothalamus, NAD^+^, Nampt, sarcopenia, skeletal muscle

## Abstract

**Background:**

Sarcopenia has become an urgent socioeconomic problem in rapidly aging societies. The pathogenesis of age‐associated sarcopenia is not fully understood and no effective therapeutic strategies have been developed to date. Recent studies have suggested the importance of the functional linkage between the brain and skeletal muscles in the pathogenesis of sarcopenia. However, the functional connections between the brain and skeletal muscles, particularly between the hypothalamus and skeletal muscles, remain unclear. In this study, we focused on the importance of nicotinamide adenine dinucleotide (NAD^+^) metabolism in the lateral hypothalamus (LH) and explored the importance of the NAD^+^‐mediated functional connection between the LH and skeletal muscle and its involvement in the pathogenesis of sarcopenia.

**Methods:**

To explore the role of NAD^+^ in the LH, we knocked down *nicotinamide phosphoribosyltransferase* (*Nampt*), a rate‐limiting enzyme in the NAD^+^ salvage pathway that is required for the maintenance of NAD^+^, by stereotaxic injection of a lentivirus encoding short hairpin RNA for *Nampt* into the LH.

**Results:**

Loss‐of‐function of *Nampt* in the LH caused decreased muscle mass (mg/cm) [tibialis anterior: 24.8 ± 0.36 vs. 22.9 ± 0.29, *p* < 0.001; gastrocnemius: 65.7 ± 1.60 vs. 60.9 ± 0.65, *p* < 0.05] and strength (mN) [382.0 ± 10.4 vs. 345.7 ± 5.47 at 100 Hz stimulation, *p* < 0.01], accompanied by disruption of the p70S6K‐S6 protein synthesis axis in skeletal muscle. Skeletal muscle of LH‐specific *Nampt*‐knockdown mice exhibited decreased levels of pyruvate and lactate, the end products of glycolysis and decreased levels of glucose metabolism‐related genes, such as *β2 adrenergic receptor* (*β2‐AR)*, *peroxisome proliferator‐activated receptor delta* (*PPARδ)*, *PPARγ* and *pyruvate dehydrogenase kinase 4* (*PDK4)*. We identified lactate as a mediator linking decreased glycolysis and protein synthesis. Lactate induces increases in intracellular Ca^2+^ levels, which induce the activation of the p70S6K‐S6 protein synthesis axis.

**Conclusions:**

Our results indicate that *Nampt* in the LH maintains skeletal muscle function by regulating lactate‐mediated Ca^2+^ signalling in skeletal muscle. Our study highlights the essential role of *Nampt* in the LH in the regulation of skeletal muscles and lactate as a mediator that links glycolysis and protein synthesis. As NAD^+^ levels in the LH decrease with age, our study provides new insights into the pathogenesis of sarcopenia.

## 1 Introduction

Sarcopenia, the age‐associated loss of muscle mass and strength, is a serious problem in rapidly aging societies [1]. Preventive and therapeutic approaches for sarcopenia are essential for extending the health span. However, molecular mechanisms underlying sarcopenia remain unclear. Therefore, only a few potential drug targets have been identified. Understanding the underlying molecular mechanisms of sarcopenia will enable us to establish therapeutic strategies to prevent sarcopenia.

Recently, the importance of nicotinamide adenine dinucleotide (NAD^+^) metabolism in age‐related pathophysiology was established [2, 3, 4, 5]. NAD^+^ is a classic coenzyme that mediates various redox reactions. Tissue/cellular NAD^+^ levels are maintained by three classical NAD^+^ biosynthesis pathways: de novo, Preiss–Handler and salvage pathways. The NAD^+^ salvage pathway, which recycles nicotinamide generated by NAD^+^‐consuming enzymes, such as sirtuins, is indispensable for the maintenance of tissue/cellular NAD^+^ levels [2]. Therefore, the pharmacological inhibition or genetic knockdown/knockout of *nicotinamide phosphoribosyltransferase* (*Nampt*), a rate‐limiting enzyme in the NAD^+^ salvage pathway, has been used to analyse the role of NAD^+^ [2, 3, 4, 5]. A progressive and systemic decline in tissue/cellular NAD^+^ levels and the resulting dysfunction of NAD^+^‐consuming enzymes accelerates age‐related pathophysiology [2, 3, 4, 5]. Therefore, supplementation with NAD^+^ intermediates, such as nicotinamide mononucleotide (NMN) or nicotinamide riboside, has been evaluated to systemically increase NAD^+^ levels and alleviate age‐related dysfunctions [6, 7].

In addition to the importance of NAD^+^ in age‐related pathophysiology, the importance of the NAD^+^‐mediated functional connection between the hypothalamus and skeletal muscle and its involvement in sarcopenia has been demonstrated [8, 9, 10]. The locomotive and metabolic functions of skeletal muscles are regulated by the central nervous system through motor neurons and sympathetic nerves [11, 12]. Recent studies have revealed the importan**ce** of the hypothalamus in the metabolic regulation of skeletal muscle and the involvement of NAD^+^‐related molecules in the hypothalamus in the pathogenesis of sarcopenia [8, 9, 10, 13, 14]. NAD^+^ levels in the lateral hypothalamus (LH), ventromedial hypothalamus (VMH) and arcuate nucleus gradually decreased during aging [15]. Overexpression of *Sirt1* in the LH and dorsomedial hypothalamus (DMH) resulted in substantial improvement in age‐related dysfunction of mitochondria in skeletal muscle [9]. Additionally, our previous study revealed the importance of *solute carrier family 12 member 8* (*Slc12a8*) in the LH in the pathogenesis of sarcopenia [10]. *Slc12a8* is an NMN transporter, an NAD^+^‐related molecule, that is highly expressed in intestinal tissues and required for NMN‐induced increases in NAD^+^ levels [16, 17]. *Slc12a8* was also expressed in the LH, and its expression gradually decreased during aging. Slc12a8‐positive cells were also localized to the LH compared with other hypothalamic nuclei, such as the DMH, VMH and arcuate [10]. Notably, knockdown of *Slc12a8* in the LH caused decreased energy expenditure, muscle mass and force. Conversely, overexpression of *Slc12a8* in the LH improved age‐associated dysfunction of skeletal muscle, suggesting that dysfunction of *Slc12a8* in the LH during aging is involved in the pathogenesis of sarcopenia [10].

NAD^+^‐related molecules in the hypothalamus are important for regulating skeletal muscle and sarcopenia pathogenesis. However, because previous studies focused on the function of upstream or downstream molecules of NAD^+^, such as *Sirt1* or *Slc12a8*, direct evaluation of the involvement of hypothalamic NAD^+^ and the molecular mechanisms by which hypothalamic NAD^+^ regulates skeletal muscle functions has not been demonstrated.

## 2 Methods

### 2.1 Animals

C57BL/6 J mice were purchased from Jackson Laboratory. All mice were housed at the institutional animal facility of the National Center for Geriatrics and Gerontology (NCGG) on a 12 h:12 h light/dark cycle with free access to water and food. All animal procedures were approved by the Experimental Animal Care and Use Committee of the NCGG (Approval number: Animal6‐1‐R1) and performed in accordance with approved guidelines. A group of 3‐ to 4‐month‐old male mice were used.

### 2.2 Cell Culture

Cells were cultured as previously described [10]. Neuro2a, C2C12, AAVpro 293T, Lenti‐X 293T and primary blasts were used. Four days after the induction of differentiation, primary or C2C12 myotubes were analysed.

To evaluate the effects of lactate, primary myotubes were pre‐treated with DMEM without fetal bovine serum for 8 h, followed by treatment with Earle's Balanced Salt Solution (EBSS) for 2 h. Primary myotubes were stimulated with lactate for 30 min and frozen in liquid nitrogen.

### 2.3 Single Muscle Fibre Isolation and Ca^2+^ Imaging Analysis

Single fibres isolation and Ca^2+^ imaging analysis was performed as previously described [18, 19]. Glucose, glucose‐6‐phosphate, fructose‐6‐phosphate, phosphoglyceric acid, phosphoenolpyruvic acid, pyruvate, lactate and *α*‐cyano‐4‐hydroxycinnamic acid (*α*CHCA) were used to assess their effects on Ca^2+^ levels.

### 2.4 Gene Knockdown by Adeno‐Associated Virus (AAV)

AAV‐DJ was used for gene knockdown in primary myotubes. Knockdown vectors were purchased from VectorBuilder. TransIT‐VirusGEN (Takara) or PEI‐max were used for AAV preparation. Three to five days after transfection, AAV was collected by freezing and thawing transfected cells. Supernatant was used as AAV solution. Primary myoblasts were infected with AAV by mixing supernatant and differentiation medium in a 1:1 ratio and induced differentiation. Four days after infection, the infected myotubes were analysed.

### 2.5 Stereotaxic Injection

Injection of lentivirus encoding short hairpin RNA (shRNA) for *Nampt* or *firefly luciferase* (*fLuc*) into the LH was performed as previously described [10]. Coordinates were as follows: relative to bregma, anterior–posterior −1.8 mm, medial‐lateral ± 0.9 mm and dorsal‐ventral −5.4 mm. Lentivirus with a titre of 1.5–2.8 × 10^10^ genome copy/mL was used. Biological and functional tests were performed 3 months after lentivirus injection. One week after stereotaxic injection, NMN (300 mg/kg/day in drinking water) was administered. For administration of NMN to the LH, 100 μM NMN was injected into the LH and tibialis anterior (TA) muscles were isolated 3 h after.

### 2.6 Measurement of Food Intake, Voluntary Activity and Body Temperature

Metabolic cage analysis was performed as previously described [10]. Food intake and voluntary running distance were measured using a running wheel and feeding monitoring system (MFD‐RQ, SHINFACTORY). After habituation for 3 days, cumulative data from 4 to 7 days was analysed. Rectal temperature at ZT13:00 was measured using KN‐91‐ad1687 (Natsume Seisakusho).

### 2.7 NAD^+^ Measurement by High Performance Liquid Chromatography (HPLC)

NAD^+^ was measured as previously described [15]. Neuro2a cells were extracted using 10% perchloric acid. Supernatant was neutralized with 3 M K_2_CO_3_, diluted with phosphate buffer and analysed using a Shimadzu HPLC system equipped with a SUPELCOSIL LC‐18‐T column (Merck).

### 2.8 Treadmill Analysis and Skeletal Muscle Force Measurement

Treadmill experiment and in vivo force measurements using 1300A (Aurora Scientific) were performed as described previously [10]. Electrically evoked contraction was induced by stimulation of the plantar flexor muscles. Stimulation with 20, 40, 60, 80, 100, 120, 150 and 200 Hz for 0.35 s with a 1 min interval was performed to evaluate the force–frequency curve. Stimulation with 100 Hz for 0.35 s was performed to evaluate tetanic force. For the fatigue resistance test, force reduction during 60 repeated stimulations with a 2 s interval at 30 Hz for 0.35 s was calculated as percentage reduction.

### 2.9 Immunohistochemical Analysis

Immunohistochemical analyses were performed as previously described [10]. Antibodies were summarized in Table S1. For quantification of Nampt signal, the average of Nampt signals in GFP‐positive areas from 2 to 12 pictures was analysed using ImageJ. KEYENCE fluorescence microscope was used for measurement of the cross‐sectional area (CSA). Approximately 1035–2537 Type 2B, 425–1101 Type 2A and 55–139 Type 1 fibres were analysed.

### 2.10 RNA Purification, cDNA Synthesis and Quantitative Polymerase Chain Reaction (qPCR) Analysis

RNA purification, cDNA synthesis and subsequent qPCR analyses were performed as described previously [10]. The primer sequences were summarized in Table S2. The expression level of each gene was normalized to that of *TATA binding protein* (*TBP)*.

### 2.11 Western Blotting and Surface Sensing of Translation (SUnSET) Analysis

Western blotting and in vivo SUnSET analysis was performed as previously described [10]. Antibodies were summarized in Table S1. For in vitro SUnSET analysis, primary myotubes were pretreated with EBSS for 4 h, then treated with EBSS with or without lactate for 4.5 h. Primary myotubes were treated with 1 μM puromycin in EBSS with or without lactate. Thirty minutes after treatment, primary myotubes were frozen in liquid nitrogen.

### 2.12 Measurement of Metabolites

Glucose tolerance tests (GTT), insulin tolerance tests (ITT) and measurement of intracellular metabolites were performed as previously described [10]. Intracellular lactate and serum insulin levels were measured using Lactate‐Glo (Promega, J5021) and insulin measurement kit (Takara, M1108), respectively. To measure the effect of formoterol, 0.2 mg/kg formoterol (Funakoshi, 15584) was administered intraperitoneally.

### 2.13 Statistical Analysis

**S**tatistical differences were assessed using Student's *t*‐test for comparison of two groups, one‐way analysis of variance with Dunnett's test or Tukey's test for comparison of multiple groups and two‐way repeated‐measures ANOVA for repeated measurements of two groups. All values are expressed as mean ± standard error of the mean. Prism 8 was used for analysis. Probabilities less than 5% (*, *p* < 0.05), 1% (**, *p* < 0.01) or 0.1% (***, *p* < 0.001), respectively, were considered to be statistically significant.

## 3 Results

### 3.1 *Nampt* in the LH Regulates Increases in Body Weight

**FIGURE 1:**
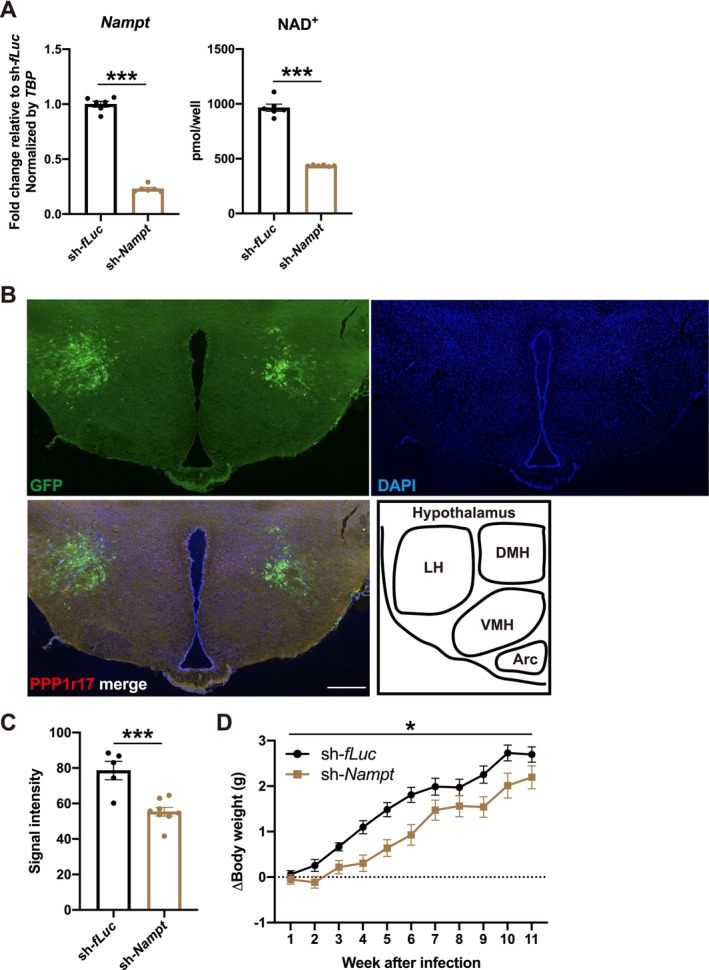
*Nampt* in the LH regulates increases of body weight. (**A**) Left: Knockdown efficiency of sh‐*Nampt* in Neuro2a cells. Right: NAD^+^ levels in *Nampt*‐knockdown Neuro2a cells. *n* = 6. (**B**) Representative fluorescent image of GFP derived from lentivirus. PPP1r17 was used as the marker of the DMH [21]. Bar: 300 μm. (**C**) Quantitative analysis for the knockdown efficiency of sh‐*Nampt* by immunohistochemistry. *n* = 5–8. (**D**) Time course changes of body weight in LH‐specific *Nampt*‐knockdown mice. *n* = 11. *p < 0.05 and ***p < 0.001 by Student's *t*‐test for (**A**) and (**C**), or by two‐way repeated‐measures ANOVA for (**D**). Error bars indicate s.e.m.

NAD^+^ levels in the LH gradually decrease during aging and previous studies have shown the functional importance of NAD^+^‐related molecules in the LH in the pathogenesis of sarcopenia [10, 15]. We therefore focused on the role of NAD^+^ in the LH in the regulation of skeletal muscle. LH plays a central role in controlling sleep, feeding behaviour and energy homeostasis [20]. To explore the role of NAD^+^ in the LH, we knocked down *Nampt* using a lentivirus encoding shRNA for *Nampt* and *Green Fluorescent Protein* (*GFP*). Lentivirus encoding shRNA for *fLuc* was used as a control. Knockdown of *Nampt* caused an approximate 80% decrease in *Nampt* RNA levels and an approximate 55% decrease in NAD^+^ levels in Neuro2a cells (Figure 1A). We then administered lentivirus to the LH by stereotaxic injection in 3–4‐month‐old mice, which caused the expression of GFP in the LH (Figure 1B). Administration of lentivirus encoding shRNA for *Nampt* caused approximately a 30% decrease in the expression of Nampt in the infected area (Figure 1C). After lentivirus administration, LH‐specific *Nampt*‐knockdown mice showed an attenuated increase in body weight (Figure 1D). This attenuated increase in body weight was rescued by NMN administration (Figure S1A), suggesting that the decreased NAD^+^ levels attenuated the increase in body weight. There were no differences in rectal temperature, food intake, or voluntary wheel‐running distance, suggesting that the decrease in body weight was not due to changes in food intake or daily activity (Figure S1B–E).

### 3.2 *Nampt* in the LH Maintains Muscle Mass and Force by Regulating Protein Synthesis

**FIGURE 2:**
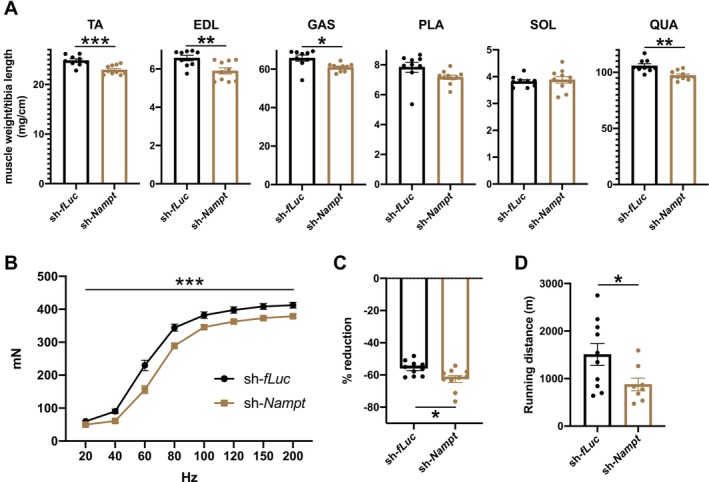
*Nampt* in the LH maintains muscle mass and force. (**A**) Hindlimb muscle weights of LH‐specific *Nampt*‐knockdown mice. *n* = 9–10. (**B**) In vivo muscle force of LH‐specific *Nampt*‐knockdown mice was shown as force‐frequency curve. *n* = 10. (**C**) Reduction of muscle force during repeated electrical stimulation in LH‐specific *Nampt*‐knockdown mice. *n* = 10. (**D**) Endurance capacity of LH‐specific *Nampt*‐knockdown mice was analysed by treadmill. *n* = 8–10. *p < 0.05, **p < 0.01 and **p < 0.01 by Student's *t*‐test for (**A**), (**C**) and (**D**), or by two‐way repeated‐measures ANOVA for (**B**). Error bars indicate s.e.m.

To evaluate the effects of *Nampt* knockdown in the LH on skeletal muscle function, we analysed hindlimb muscle weight 3 months after lentivirus administration. Because LH‐specific *Nampt*‐knockdown mice showed changes in body weight, we normalized muscle weight to tibial length (Figure 2A). LH‐specific *Nampt*‐knockdown mice showed decreased muscle weight in fast muscles, including the TA, gastrocnemius (GAS), plantaris (PLA) and quadriceps (QUA) muscles but not in the soleus (SOL) slow muscle (Figure 2A). In addition to tibial length, QUA muscle weight was normalized by femoral length (Figure S2A). When muscle weights were normalized to body weight, we did not observe any differences in fast muscle weights in LH‐specific *Nampt*‐knockdown mice, suggesting that a decrease in muscle weight is one of the causes of decreased body weight in LH‐specific *Nampt*‐knockdown mice (data not shown). The decreased TA and GAS muscle weights in LH‐specific *Nampt*‐knockdown mice were rescued by NMN (Figure S2B). Furthermore, we did not observe any differences in epididymal white adipose tissue weight in LH‐specific *Nampt*‐knockdown mice (Figure S2C). We also analysed the CSA of the GAS muscle by staining with antibodies against myosin heavy chain (MyHC) Type IIA, Type IIB and Type I (Figure S2D). LH‐specific *Nampt*‐knockdown mice showed decreased CSA of Type IIA and Type IIB fast muscle fibres (Figure S2D–F). In contrast, there was no difference in the CSA of Type I slow muscle fibres (Figure S2D,G). These results indicated that *Nampt* in the LH is important for the maintenance of fast muscle mass.

We then explored the effect of *Nampt* knockdown in the LH on muscle force. Knockdown of *Nampt* in the LH caused a decrease in skeletal muscle forces during high‐frequency electrical stimulation, indicating that *Nampt* in the LH is essential for the maintenance of muscle force (Figure 2B). The reduced tetanic force in LH‐specific *Nampt*‐knockdown mice was rescued by NMN, suggesting that the decreased NAD^+^ levels reduced muscle force in LH‐specific *Nampt*‐knockdown mice (Figure S2H). LH‐specific *Nampt*‐knockdown mice also showed a significant reduction in muscle force after repeated electrical stimulation, suggesting the reduction of fatigue resistance in these mice (Figure 2C). We then performed treadmill analysis to assess endurance capacity. Control mice ran approximately 1500 m (Figure 2D). Contrary, LH‐specific *Nampt*‐knockdown mice showed an approximately 40% decrease in running distance (Figure 2D). Collectively, our results indicate that *Nampt* in the LH is essential for maintaining muscle weight and force.

**FIGURE 3:**
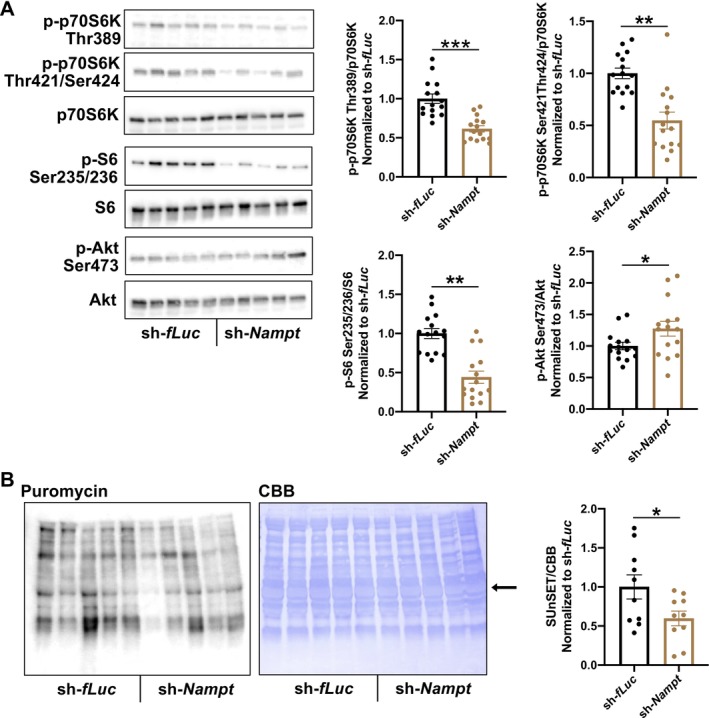
*Nampt* in the LH regulates protein synthesis in skeletal muscle. (**A**) Left: Representative western blot images showing phosphorylation of p70S6K, S6 and Akt of TA muscles from LH‐specific *Nampt*‐knockdown mice. Right: Quantification for the phosphorylation of p70S6K, S6 and Akt in LH‐specific *Nampt*‐knockdown mice. *n* = 15. (**B**) Representative western blot images for puromycin labelled‐peptides (left) of LH‐specific *Nampt*‐knockdown mice. CBB staining was shown as control (middle). Right: Quantification of puromycin labelled‐peptides. Signals around 65 kDa (Arrow) were analysed. *n* = 10. *p < 0.05, **p < 0.01 and ***p < 0.001 by Student's *t*‐test. Error bars indicate s.e.m.

Muscle mass is regulated by the balance between protein synthesis and degradation [22]; therefore, we evaluated the signalling molecules that regulate protein synthesis and degradation. LH‐specific *Nampt*‐knockdown mice exhibited attenuated phosphorylation of p70S6K, a central molecule that regulates protein synthesis (Figures 3A and S3A). LH‐specific *Nampt*‐knockdown mice also exhibited decreased phosphorylation of S6, a downstream target of p70S6K (Figure 3A). To measure protein synthesis, we perform SUnSET analysis. LH‐specific *Nampt*‐knockdown mice showed decreased amounts of puromycin‐labelled newly synthesized peptides, indicating that *Nampt* in the LH is required for the maintenance of protein synthesis (Figure 3B).

Akt is a well‐known upstream regulator of protein synthesis [22]. In contrast to the phosphorylation of p70S6K and S6, Akt phosphorylation was increased in LH‐specific *Nampt*‐knockdown mice, suggesting that the decreased phosphorylation of p70S6K and S6 was not due to the downregulation of Akt (Figure 3A). Furthermore, to evaluate the direct effect of NMN on p70S6K in skeletal muscle, we administered NMN to the LH and measured the phosphorylation of p70S6K in skeletal muscle. However, no change was observed after NMN injection (Figure S3B). Taken together, *Nampt* in the LH maintains muscle weight by regulating protein synthesis.

### 3.3 *Nampt* in the LH Regulates Glycolysis in Skeletal Muscle

**FIGURE 4:**
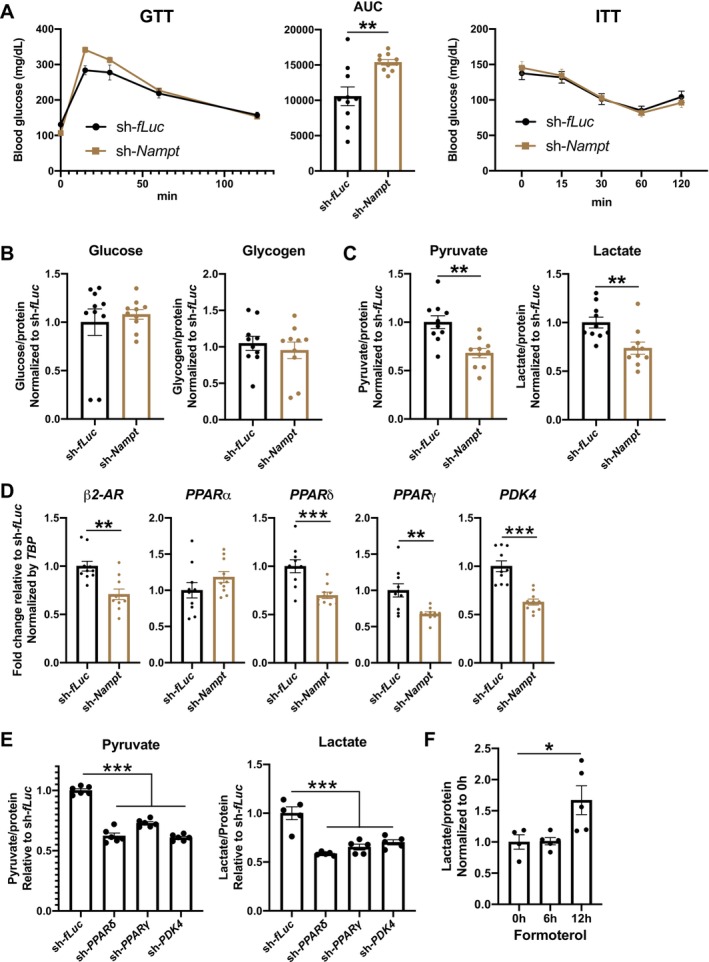
*Nampt* in the LH regulates glycolysis in skeletal muscle. (**A**) Left: Glucose tolerance test in LH‐specific *Nampt*‐knockdown mice. Middle: Quantification of area under the curve (AUC) of glucose tolerance test in LH‐specific *Nampt*‐knockdown mice. Right: Insulin tolerance test in LH‐specific *Nampt*‐knockdown mice. *n* = 10. (**B**) Glucose and glycogen levels in TA muscle of LH‐specific *Nampt*‐knockdown mice. *n* = 10. (**C**) Pyruvate and lactate levels in TA muscle of LH‐specific *Nampt*‐knockdown mice. *n* = 10. (**D**) Expression of *β2‐AR*, *PPARα*, *PPARδ*, *PPARγ* and *PDK4* in TA muscle of LH‐specific *Nampt*‐knockdown mice. *n* = 10. (**E**) Pyruvate and lactate levels in *PPARδ*‐, *PPARγ*‐ or *PDK4*‐knockdown myotubes. *n* = 5. (**F**) Time course changes of lactate levels in TA muscle after administration of formoterol. *n* = 4–5. *p < 0.05, **p < 0.01 and ***p < 0.001 by Student's *t*‐test for (**A**), (**C**) and (**D**), or one‐way ANOVA with Dunnett's test for (**E**) and (**F**). Error bars indicate s.e.m.

Because glucose metabolism is a fundamental energy source during exercise and force production, we analysed glucose metabolism‐related metabolites and genes in skeletal muscle. GTT and ITT were performed to evaluate systemic glucose metabolism. After glucose administration, the maximum blood glucose levels were higher in LH‐specific *Nampt*‐knockdown mice (Figure 4A). We measured serum insulin levels at ZT13:00 and after 24 h fasting. LH‐specific *Nampt*‐knockdown mice showed decreased insulin levels at ZT13:00 (Figure S4A), suggesting the changes in insulin levels were one of the causes of GTT changes in these mice. Contrary, there were no differences in ITT, suggesting that systemic insulin sensitivity was not affected in LH‐specific *Nampt*‐knockdown mice (Figure 4A). We then analysed glucose metabolism‐related metabolites in skeletal muscle. There were no differences in the levels of glycogen and glucose in the TA muscles of LH‐specific *Nampt*‐knockdown mice (Figure 4B). However, the amount of pyruvate, the end product of glycolysis and lactate, produced from pyruvate by Lactate Dehydrogenase A (LDHA), were significantly decreased in LH‐specific *Nampt*‐knockdown mice, suggesting decreased glycolysis in these mice (Figure 4C). We also analysed glucose metabolism‐related genes in skeletal muscle. LH‐specific *Nampt*‐knockdown mice showed decreased expression of *β2 adrenergic receptor* (*β2‐AR*), *peroxisome proliferator‐activated receptor delta* (*PPARδ*), *PPARγ* and *Pyruvate dehydrogenase kinase 4* (*PDK4*) (Figure 4D). There was no difference in the expression of *PPARα*. The expression of *PPARδ*, *PPARγ* and *PDK4* is induced by the activation of *β2‐AR* [10, 23, 24]. *PPARδ* and *PPARγ* are important regulators of glucose metabolism in skeletal muscle because activation of *PPARδ* and *PPARγ* causes increased glucose uptake and utilization [25, 26, 27, 28, 29]. *PDK4*, which regulates pyruvate dehydrogenase to control the balance between glucose and fatty acid utilization, is also important for glycolysis because insulin‐induced uptake of glucose and basal levels of glucose‐6‐phosphate, an intermediary metabolite of glycolysis, were decreased in *PDK2*/*4* double‐knockout mice [30, 31]. To evaluate the effects of decreased expression of *PPARδ*, *PPARγ* and *PDK4* on the amount of pyruvate and lactate, we knocked down *PPARδ*, *PPARγ* or *PDK4* in primary myotubes using AAV. Knockdown efficiencies of sh‐*PPARδ*, sh‐*PPARγ* and sh‐*PDK4* were approximately 92%, 87% and 90%, respectively (Figure S4B). Knockdown of *PPARδ*, *PPARγ* or *PDK4* caused the decreased amounts of pyruvate and lactate, suggesting that the decreased expression of *PPARδ*, *PPARγ* and *PDK4* caused decreased pyruvate and lactate in LH‐specific *Nampt*‐knockdown mice (Figure 4E). Furthermore, the administration of formoterol, a *β2‐AR* agonist that induces the expression of *PPARδ*, *PPARγ* and *PDK4* [10], increased lactate levels in skeletal muscle (Figure 4F), suggesting that lactate levels are regulated by *β2‐AR* and its downstream *PPARδ*, *PPARγ* and *PDK4*. We also evaluated the effects of formoterol on the expression of *PPARδ*, *PPARγ* and *PDK4* in LH‐specific *Nampt*‐knockdown mice. These mice showed attenuated formoterol‐induced increases in *PPARδ*, *PPARγ* and *PDK4* (Figure S4C), suggesting the dysregulated activation of *β2‐AR* in LH‐specific *Nampt*‐knockdown mice. Taken together, *Nampt* in the LH regulates glycolysis by regulating *β2‐AR*, *PPARδ*, *PPARγ* and *PDK4*.

### 3.4 Lactate‐Mediated Ca^2+^ Signalling as an Upstream Regulator of the p70S6K‐S6 Axis

**FIGURE 5:**
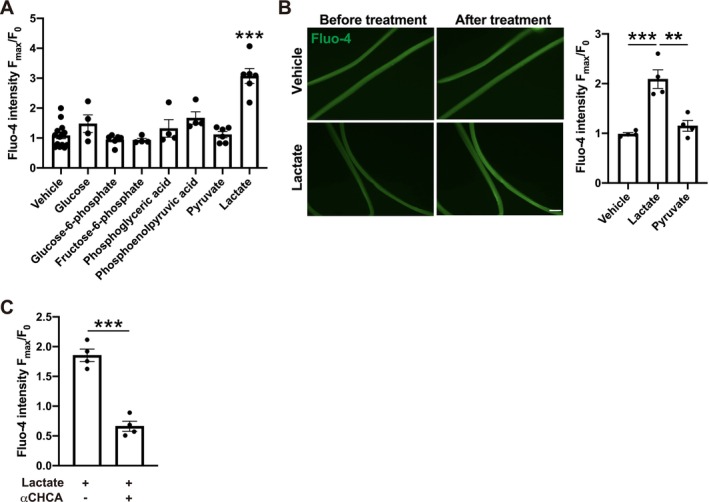
Lactate induces increases in intracellular Ca^2+^ levels. (**A**) Changes of Fluo‐4 intensity after treatment with glycolysis‐related metabolites in C2C12 myotubes. *n* = 4–14. (**B**) Left: Representative fluorescent Fluo‐4 images of single muscle fibres before and after treatment with lactate. Bar: 100 μm. Right: Quantification analysis of Fluo‐4 intensity in single muscle fibres after treatment with lactate or pyruvate. *n* = 4. (**C**) Lactate‐induced increases of Fluo‐4 intensity was inhibited by co‐treatment with *α*CHCA. *n* = 4. **p < 0.01 and ***p < 0.001 by Student's *t*‐test for (**C**), one‐way ANOVA with Dunnett's test for (**A**), or one‐way ANOVA with Tukey's test for (**B**). Error bars indicate s.e.m.

Our results indicated that both glycolysis and protein synthesis were disrupted in LH‐specific *Nampt*‐knockdown mice (Figures 3 and 4). To evaluate the hierarchical relationship between glycolysis and protein synthesis, we focused on the role of glycolysis‐related metabolites in Ca^2+^ signalling. In previous studies, we demonstrated the essential role of Ca^2+^ signalling as an upstream regulator of the p70S6K‐S6 axis [18, 19]. Increases in intracellular Ca^2+^ levels by exercise, mechanical load or chemical stimulation cause activation of the p70S6K‐S6 axis and induce muscle hypertrophy [18, 19]. We hypothesized that we could identify the essential metabolites that link glycolysis and the p70S6K‐S6 axis by analysing their effects on intracellular Ca^2+^ levels. We treated C2C12 myotubes with glycolysis‐related metabolites and analysed their effects on intracellular Ca^2+^ levels. We found that intracellular Ca^2+^ levels were increased by lactate (Figure 5A). Lactate‐induced increases in Ca^2+^ levels were also observed in primary myotubes and single muscle fibres, indicating that lactate increases Ca^2+^ levels in both immature myotubes and adult muscle fibres (Figures 5B and S5A). Pyruvate did not induce an increase in Ca^2+^ levels in myotubes or single muscle fibres (Figure 5B). Lactate induced an increase in Ca^2+^ levels in dose‐dependent manner in C2C12 myotubes (Figure S5B). Furthermore, these increases in Ca^2+^ levels were inhibited by *α*‐CHCA, an inhibitor for monocarboxylate transporters that is required for the uptake of lactate both in C2C12 myotubes and single fibres, suggesting that an increase in intracellular lactate is required for the increases in Ca^2+^ levels (Figures 5C and S5C).

**FIGURE 6:**
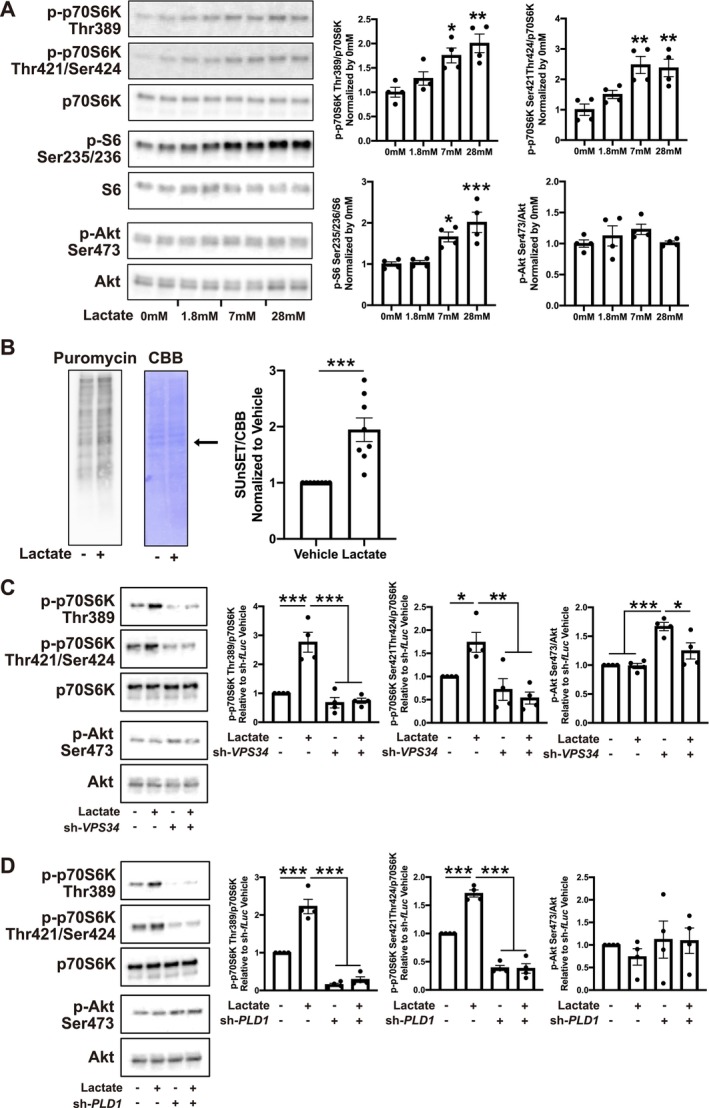
Lactate induces activation of p70S6K‐S6 axis through class III PI3K. (**A**) Left: Representative images of western blot analysis showing phosphorylated and total p70S6K, S6 and Akt of lactate‐treated primary myotubes. Right: Quantification for the phosphorylation of p70S6K, S6 and Akt in lactate‐treated primary myotubes. *n* = 4. (**B**) Representative western blot images for puromycin labelled‐peptides (left) and CBB staining (middle) of lactate‐treated primary myotubes. Right: Quantitative analysis of puromycin labelled‐peptides. Signals around 50 kDa (Arrow) were quantified. *n* = 8. (**C**) Left: Representative western blot analysis showing phosphorylated and total p70S6K and Akt of lactate‐treated *VPS34*‐knockdown primary myotubes. Right: Quantification for the phosphorylation levels of p70S6K, S6 and Akt in lactate‐treated *VPS34*‐knockdown primary myotubes. *n* = 4. (**D**) Left: Representative Western blot analysis showing phosphorylated and total p70S6K and Akt of lactate‐treated *PLD1*‐knockdown primary myotubes. Right: Quantification for the phosphorylation levels of p70S6K, S6 and Akt in lactate‐treated *PLD1*‐knockdown primary myotubes. *n* = 4. *p < 0.05, **p < 0.01 and ***p < 0.001 by Student's *t*‐test for (**B**), one‐way ANOVA with Dunnett's test for (**A**), or one‐way ANOVA with Tukey's test for (**C**) and (**D**). Error bars indicate s.e.m.

**FIGURE 7:**
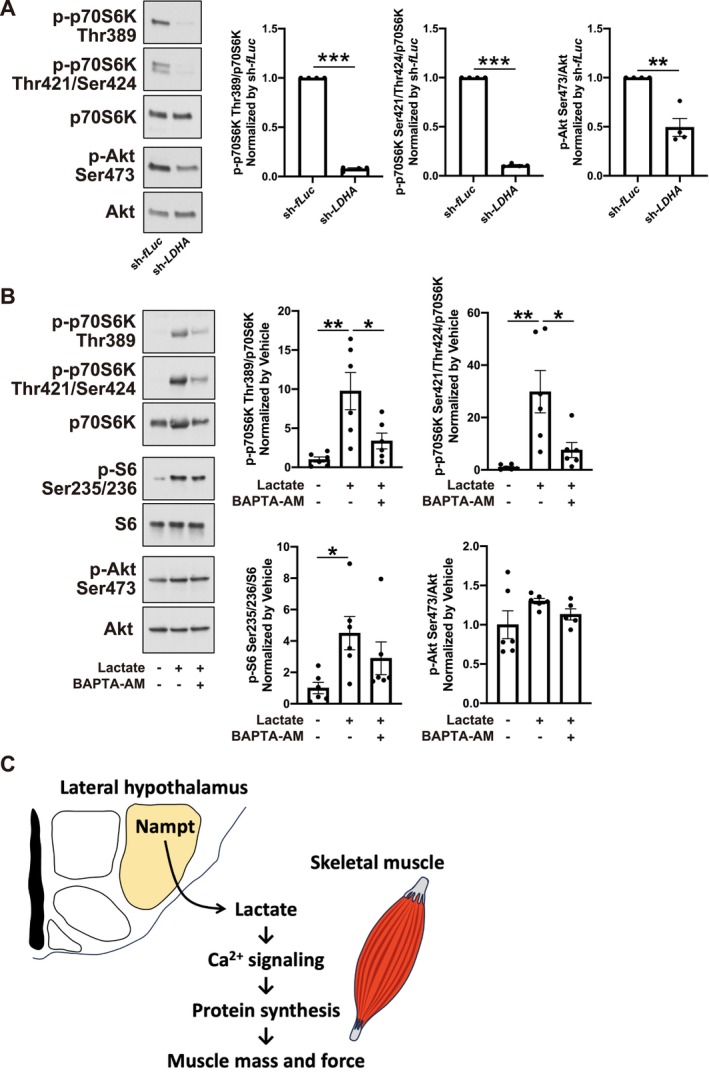
Lactate‐mediated Ca^2+^ signalling as an upstream regulator of p70S6K‐S6 axis. (**A**) Left: Representative Western blot analysis showing phosphorylated and total p70S6K and Akt of *LDHA*‐knockdown primary myotubes. Right: Quantification for the phosphorylation levels of p70S6K, S6 and Akt in lactate‐treated *LDHA*‐knockdown primary myotubes. *n* = 4. (**B**) Left: Representative Western blot analysis showing phosphorylated and total p70S6K, S6 and Akt of lactate‐ and/or BAPTA‐AM‐injected TA muscle. Right: Quantitative analysis for the phosphorylation levels of p70S6K, S6 and Akt in lactate‐ and/or BAPTA‐AM‐injected TA muscle. *n* = 5–6. (**C**) A scheme for the Nampt‐mediated regulation of skeletal muscle. *p < 0.05, **p < 0.01 and ***p < 0.001 by Student's *t*‐test for (**A**) and one‐way ANOVA with Tukey's test for (**B**). Error bars indicate s.e.m.

We then evaluated the effects of lactate on the p70S6K‐S6 axis. Treatment with lactate caused phosphorylation of p70S6K and S6 in a dose‐dependent manner (Figures 6A and S6A). Under the same conditions, Akt phosphorylation did not change, suggesting that lactate activated the p70S6K‐S6 axis in an Akt‐independent manner (Figure 6A). In addition to the activation of the p70S6K‐S6 axis, treatment with lactate increased protein synthesis (Figure 6B). In previous studies, we showed that increases in Ca^2+^ levels caused phosphorylation of p70S6K through class III phosphatidylinositol‐3 kinase (PI3K) Vps34 in an Akt‐ and class I PI3K‐independent manner [32]. To assess the involvement of class III PI3K and its downstream phospholipase D1 (PLD1), we knocked down *Vps34* and *PLD1* (Figure S6B). Lactate‐induced phosphorylation of p70S6K was prevented by knockdown of *Vps34* or *PLD1*, indicating that lactate‐induced phosphorylation of p70S6K is mediated by class III PI3K (Figures 6C,D and S6C,D). We also analysed the relationship between intracellular lactate levels and p70S6K phosphorylation by knockdown of *LDHA* (Figure S7A). Knockdown of *LDHA* resulted in decreased intracellular lactate levels and caused decreased p70S6K phosphorylation (Figures 7A and S7B,C). In contrast to lactate treatment, knockdown of *LDHA* caused decreased Akt phosphorylation, suggesting that basal intracellular lactate levels maintain p70S6K activity in an Akt‐dependent manner. Finally, we evaluated the effect of lactate and its relationship with intracellular Ca^2+^ levels in vivo by co‐administration with the intracellular Ca^2+^ chelator BAPTA‐AM. Administration of lactate caused phosphorylation of p70S6K and S6, which was prevented by co‐administration of BAPTA‐AM, suggesting that lactate activates the p70S6K‐S6 axis by activating Ca^2+^ signalling (Figures 7B and S7D). Lactate‐induced phosphorylation of p70S6K was maintained in LH‐specific *Nampt*‐knockdown mice, suggesting that the sensitivity for lactate was not affected in these mice (Figure S7E). Because intracellular lactate levels were decreased in LH‐specific *Nampt*‐knockdown mice (Figure 4C), these results indicate that *Nampt* in the LH maintains skeletal muscle function through lactate‐mediated Ca^2+^ signalling in skeletal muscle (Figure 7C).

## 4 Discussion

In this study, we demonstrated the importance of *Nampt* in the LH in the regulation of skeletal muscles. Knockdown of *Nampt* in the LH caused a decrease in muscle mass and force, accompanied by disrupted glycolysis and protein synthesis. Furthermore, the disruption of glycolysis and protein synthesis was linked to lactate‐mediated Ca^2+^ signalling, suggesting a functional link between metabolic flexibility and mechanical properties (Figure 7C).

This study focused on the functional linkage between brain and skeletal muscle by analysing *Nampt* in the LH and showed that the reduction of NAD^+^ in the LH during aging contributes to the pathogenesis of sarcopenia. Notably, the decreased cognitive function, including dementia, is a risk factor for sarcopenia [33]. In contrast, decreased physical function is significantly associated with decreased cognitive function and a higher risk of dementia, suggesting a reciprocal relationship between age‐related dysfunction in brain and skeletal muscles [34]. However, the functional connections between brain and skeletal muscles, particularly between the hypothalamus and skeletal muscles, remain unclear. NAD^+^ levels in the arcuate nucleus, VMH and LH gradually decreased during aging [15]. The present study highlights the importance of NAD^+^ in the LH for the regulation of skeletal muscle and suggests that decreased NAD^+^ levels in the LH during aging are involved in the pathogenesis of sarcopenia. Notably, an association between hypothalamic Alzheimer's disease pathology and body mass index in humans was established [35]. The degree of amyloid plaques in the LH and tuberomammillary nucleus was associated with a lower body mass index while alive [35], which is similar to our observation in LH‐specific *Nampt*‐knockdown mice (Figure 1D). Further research is needed to understand how *Nampt* or NAD^+^ in the LH regulates skeletal muscles. Because decreases in NAD^+^ levels in other hypothalamic nuclei, such as the arcuate and VMH were also observed during aging [15], the relationship between sarcopenia and reduced NAD^+^ levels in other hypothalamic nuclei including the DMH, VMH and arcuate also needs to be clarified.

We observed decreased body weight in LH‐specific *Nampt*‐knockdown mice (Figure 1D). In addition to decreased muscle weight, we observed slight but not statistically significant, decreases in food intake (Figure S1C). Because we only analysed food intake for 5 days, the slight decreases in food intake for longer periods may affect body weight. A more precise measurement of food intake for longer periods is required to identify the causes of decreased body weight in LH‐specific *Nampt*‐knockdown mice.

In a previous study, we showed that *Slc12a8* in the LH regulates skeletal muscle through sympathetic nerve [10]. The major function of sympathetic innervation in skeletal muscles is considered to be blood flow control. However, direct innervation of sympathetic nerves to skeletal muscle fibres was reported [11, 12]. Although the function of sympathetic nerve innervation of skeletal muscle fibres is still under investigation, previous studies have suggested that sympathetic nerves regulate skeletal muscle glycolysis via *β2‐AR* [10, 23, 24]. LH‐specific *Nampt*‐knockdown mice showed decreased levels of pyruvate and lactate, accompanied by decreased expression of *β2‐AR*, *PPARδ*, *PPRAγ* and *PDK4* (Figure 4C,D). Notably, administration of formoterol causes upregulation of *PPARδ*, *PPARγ* and *PDK4* in skeletal muscle [10, 23, 24]. Furthermore, the administration of clenbuterol, an agonist of *β2‐AR*, increased lactate levels in skeletal muscle, accompanied by muscle hypertrophy [36]. We observed similar increases in lactate levels by formoterol and formoterol‐induced increases in *PPARδ*, *PPARγ* and PDK4 were attenuated in LH‐specific *Nampt*‐knockdown mice (Figure S4C), suggesting that decreased *β2‐AR* signalling under the control of the LH dysregulated glycolysis and decreased muscle mass in LH‐specific *Nampt*‐knockdown mice. Of note, we observed decreased running distance in LH‐specific *Nampt*‐knockdown mice, but not in voluntary running distance (Figures S1E and 2D). The reduction in muscle weight or force in these mice was approximately 5%–10% (Figure 2A,B). This mild reduction in muscle function may not affect voluntary running distance. These results suggested that *β2‐AR* signalling is required for the maintenance of maximum muscle force. The molecular mechanisms that link NAD^+^ in the LH and *β2‐AR* in skeletal muscle should be clarified in future studies. In addition to the *β2‐AR*, our study suggested that systemic changes in insulin levels also contributed to the decreased systemic glucose tolerance in LH‐specific *Nampt*‐knockdown mice (Figure S4A). Although phosphorylation of Akt increased in LH‐specific *Nampt*‐knockdown mice, these results suggested that both the sympathetic nervous system and the endocrine system underlie the phenotype of LH‐specific *Nampt*‐knockdown mice.

The role of lactate in regulating muscle mass was reported by several studies [37, 38, 39]. Administration of lactate causes p70S6K phosphorylation and promotes muscle hypertrophy, similar to our observations. Our study suggests that the effects of lactate on skeletal muscle are mediated by Ca^2+^ signalling. In addition to lactate, Ca^2+^ signalling is activated by several physiological stimuli that induce the activation of the p70S6K–S6 axis and promote muscle hypertrophy [18, 19]. Our present results suggest that *Nampt* and NAD^+^ in the LH regulate the amount of lactate in skeletal muscle and that lactate‐mediated Ca^2+^ signalling intermediates the functional connection between the LH and skeletal muscle. Because lactate levels fluctuate with various stimuli, including exercise and nutritional changes, various signals from inside and outside skeletal muscle are integrated as the amount of lactate to maintain adequate muscle mass and strength, and the disruption of these integrated signals contributes to the pathogenesis of sarcopenia. Interestingly, LH‐specific *Slc12a8*‐knockdown mice showed decreased lactate levels in skeletal muscle (data not shown), suggesting a common molecular mechanism for decreased muscle function in LH‐specific *Slc12a8*‐ and *Nampt*‐knockdown mice.

Although our study indicates the importance of lactate in the regulation of Ca^2+^ signalling, the molecular mechanisms by which lactate regulates intracellular Ca^2+^ levels have not been elucidated. Lactate activates the inositol trisphosphate receptor (IP_3_R), which is essential for Ca^2+^ release from the endoplasmic reticulum in colon cancer cells [40]. We explored the involvement of IP_3_R in the lactate‐induced increase in Ca^2+^ levels. However, xestospongin C, an inhibitor of IP_3_R, did not affect the lactate‐induced increase in Ca^2+^ levels (data not shown). Using a genetic model, the contributions of IP_3_R and other Ca^2+^ channels should be examined to identify the targets of lactate. Furthermore, although we elucidated the important role of lactate in Ca^2+^ signalling, we did not evaluate the effects of lactate on other metabolic pathways. Because the effect of lactate on Ca^2+^ signalling is possibly mediated by other metabolic systems, such as mitochondria, the effects of lactate on other metabolic pathways should be analysed in future studies.

Although we showed the importance of *Nampt* in the LH for the regulation of skeletal muscle, more complex metabolic and molecular dysfunctions should underlie the pathogenesis of sarcopenia because NAD^+^ decreases in other hypothalamic nuclei during aging [15]. The contribution of *Nampt* in the LH to sarcopenia also needs to be investigated by overexpression of *Nampt* in the LH of aged mice in future studies. In addition, we used only male mice and did not evaluate sex‐dependent differences. A comparative study to analyse the sex‐dependent differences in *Nampt* in the LH for the regulation of skeletal muscle should be performed in future. Furthermore, we could not identify which types of cells contributed to the LH‐skeletal muscle axis. In addition to NAD^+^ levels in neurons, the contribution of NAD^+^ in the other cell types, such as oligodendrocytes or astrocytes, also needs to be clarified in future studies.

## Conflicts of Interest

NMN was obtained from Kyowa Hakko Bio CO. LTD.

## Supporting information


**Figure S1:** **Related to Figure 1,** *Nampt* **in the LH regulates increases of body weight** (**A**) Effect of NMN on body weight in LH‐specific *Nampt*‐knockdown mice. *n* = 10. (**B**) Rectal temperature of LH‐specific *Nampt*‐knockdown mice. *n* = 14–15. (**C**) Average food intake during ZT0:00–12:00 (left) or ZT12:00–24:00 (right) in LH‐specific *Nampt*‐knockdown mice. *n* = 8. (**D**) Accumulated food intake for 5 days in LH‐specific *Nampt*‐knockdown mice. *n* = 8. (**E**) Average running distance by running wheel during ZT0:00–12:00 (left) or ZT12:00–24:00 (right) in LH‐specific *Nampt*‐knockdown mice. *n* = 8. *p < 0.05 and ***p < 0.001 by Tukey's test for (**A**). Error bars indicate s.e.m.
**Figure S2: Related to Figure 2,** *Nampt* **in the LH maintains muscle mass and force in skeletal muscle** (**A**) QUA muscle weights of LH‐specific *Nampt*‐knockdown mice normalized by femoral length. *n* = 9. (**B**) Changes of TA and GAS muscle weights in LH‐specific *Nampt*‐knockdown mice with or without NMN administration. *n* = 10. (**C**) Epididymal white adipose tissue weights of LH‐specific *Nampt*‐knockdown mice. *n* = 9–10. (**D**) Representative image for Type 2A (left, red), Type 2B (middle, red) and Type 1 fibres (right, red). Type 2B fibres were co‐stained with laminin α2 (middle, green) and DAPI (middle, blue). Bar: 100 μm. (**E**‐**G**) Left: The cross‐sectional areas of Type2A fibre (**E**), Type2B fibre (**F**) or Type 1 fibre (**G**). Right: The average cross‐sectional areas of Type2A fibre (**E**), Type2B fibre (**F**) or Type 1 fibre (**G**). *n* = 5. (**H**) In vivo tetanic force of LH‐specific *Nampt*‐knockdown mice with or without NMN administration. *n* = 10. *p < 0.05 and **p < 0.01 by Student' s *t*‐test for (**A**), (**E**) and (**F**) or by One‐way ANOVA with Tukey's test for (**B**) and (**H**). Error bars indicate s.e.m.
**Figure S3: Related to Figure 3,** *Nampt* **in the LH regulates protein synthesis in skeletal muscle** (**A**) Representative CBB image of Figure 3A. (**B**) Left: Representative Western blot analysis showing phosphorylated and total p70S6K in TA muscle of NMN‐injected mice. Right: Quantitative analysis for the phosphorylation levels of p70S6K in TA muscle of NMN‐injected mice. *n* = 5.
**Figure S4: Related to Figure 4,** *Nampt* **in the LH regulates glycolysis in skeletal muscle** (**A**) Serum insulin levels at ZT 13:00 and after 24 h after fasting in LH‐specific *Nampt*‐knockdown mice. *n* = 9. (**B**) Knockdown efficiency of sh‐*PPARδ*, sh‐*PPARγ* or sh‐*PDK4* in primary myotubes. *n* = 6. (**C**) Expression of *PPARδ*, *PPARγ* and *PDK4* in TA muscle of formoterol‐injected LH‐specific *Nampt*‐knockdown mice. *n* = 7–9. *p < 0.05, **p < 0.01 and ***p < 0.001 by Tukey's test for (**A**) and (**C**), or Student' s *t*‐test for (**B**). Error bars indicate s.e.m.
**Figure S5: Related to Figure 5, Lactate induces increases in intracellular Ca^2^^+^ levels.** (**A**) Representative fluorescent Fluo‐4 images of primary myotubes before and after treatment with lactate. Bar: 100 μM. (**B**) Dose dependent changes of Fluo‐4 intensity in lactate‐treated C2C12 myotubes. *n* = 6. (**C**) Lactate‐induced increases of Fluo‐4 intensity was inhibited by co‐treatment with *α*CHCA. *n* = 6. ***p < 0.001 by one‐way ANOVA with Dunnett' s test for (**B**), or one‐way ANOVA with Tukey's test for (**C**). Error bars indicate s.e.m.
**Figure S6: Related to Figure 6, Lactate induces activation of p70S6K‐S6 axis through class III PI3K** (**A**) Representative CBB image for Figure 6A. (**B**) Knockdown efficiency of sh‐*VPS34* or sh‐*PLD1* in primary myotubes. *n* = 3. (**C–D**) Representative CBB image for Figure 6C and 6D. ***p < 0.001 by Student’ s t‐test. Error bars indicate s.e.m.
**Figure S7: Related to Figure 7, Lactate‐mediated Ca^2^^+^ signalling as an upstream regulator of p70S6K‐S6 axis.** (**A**) Knockdown efficiency of sh‐*LDHA* in primary myotubes. *n* = 4. (**B**) Lactate levels in *LDHA*‐knockdown primary myotubes. *n* = 5. (**C–D**) Representative CBB image for Figure 7A and 7B. (**E**) Left: Representative Western blot analysis showing phosphorylated and total p70S6K in lactate‐injected TA muscle of LH‐specific *Nampt*‐knockdown mice. Right: Quantitative analysis for the phosphorylation levels of p70S6K in lactate‐injected TA muscle of LH‐specific *Nampt*‐knockdown mice. *n* = 9. *p < 0.05, **p < 0.01 and ***p < 0.001 by Student' s *t*‐test for (**A**) and (**B**), or one‐way ANOVA with Tukey's test for (**E**). Error bars indicate s.e.m.
**Table S1:** Supporting information.
**Table S2:** Supporting information.
