# Supplementary material for: Nicotinamide phosphoribosyltransferase (Nampt) in Lateral Hypothalamus Maintains Skeletal Muscle Functions Through Lactate‐Mediated Calcium Signalling in Male Mice
Source: J Cachexia Sarcopenia Muscle. 2025 Sep 16;16(5):e70055. doi: 10.1002/jcsm.70055 (PMC12439195; doi:10.1002/jcsm.70055)
Supplement: Supplementary file 1 — Figure S1: Related to Figure 1, Nampt in the LH regulates increases of body weight (A) Effect of NMN on body weight in LH‐specific Nampt‐knockdown mice. n = 10. (B) Rectal temperature of LH‐specific Nampt‐knockdown mice. n = 14–15. (C) Average food intake during ZT0:00–12:00 (left) or ZT12:00–24:00 (right) in LH‐specific Nampt‐knockdown mice. n = 8. (D) Accumulated food intake for 5 days in LH‐specific Nampt‐knockdown mice. n = 8. (E) Average running distance by running wheel during ZT0:00–12:00 (left) or ZT12:00–24:00 (right) in LH‐specific Nampt‐knockdown mice. n = 8. *p < 0.05 and ***p < 0.001 by Tukey's test for (A). Error bars indicate s.e.m. Figure S2: Related to Figure 2, Nampt in the LH maintains muscle mass and force in skeletal muscle (A) QUA muscle weights of LH‐specific Nampt‐knockdown mice normalized by femoral length. n = 9. (B) Changes of TA and GAS muscle weights in LH‐specific Nampt‐knockdown mice with or without NMN administration. n = 10. (C) Epididymal white adipose tissue weights of LH‐specific Nampt‐knockdown mice. n = 9–10. (D) Representative image for Type 2A (left, red), Type 2B (middle, red) and Type 1 fibres (right, red). Type 2B fibres were co‐stained with laminin α2 (middle, green) and DAPI (middle, blue). Bar: 100 μm. (E‐G) Left: The cross‐sectional areas of Type2A fibre (E), Type2B fibre (F) or Type 1 fibre (G). Right: The average cross‐sectional areas of Type2A fibre (E), Type2B fibre (F) or Type 1 fibre (G). n = 5. (H) In vivo tetanic force of LH‐specific Nampt‐knockdown mice with or without NMN administration. n = 10. *p < 0.05 and **p < 0.01 by Student' s t‐test for (A), (E) and (F) or by One‐way ANOVA with Tukey's test for (B) and (H). Error bars indicate s.e.m. Figure S3: Related to Figure 3, Nampt in the LH regulates protein synthesis in skeletal muscle (A) Representative CBB image of Figure 3A. (B) Left: Representative Western blot analysis showing phosphorylated and total p70S6K in TA muscle of NMN‐injected mice. Right: Quanti [file JCSM-16-e70055-s001.pdf]

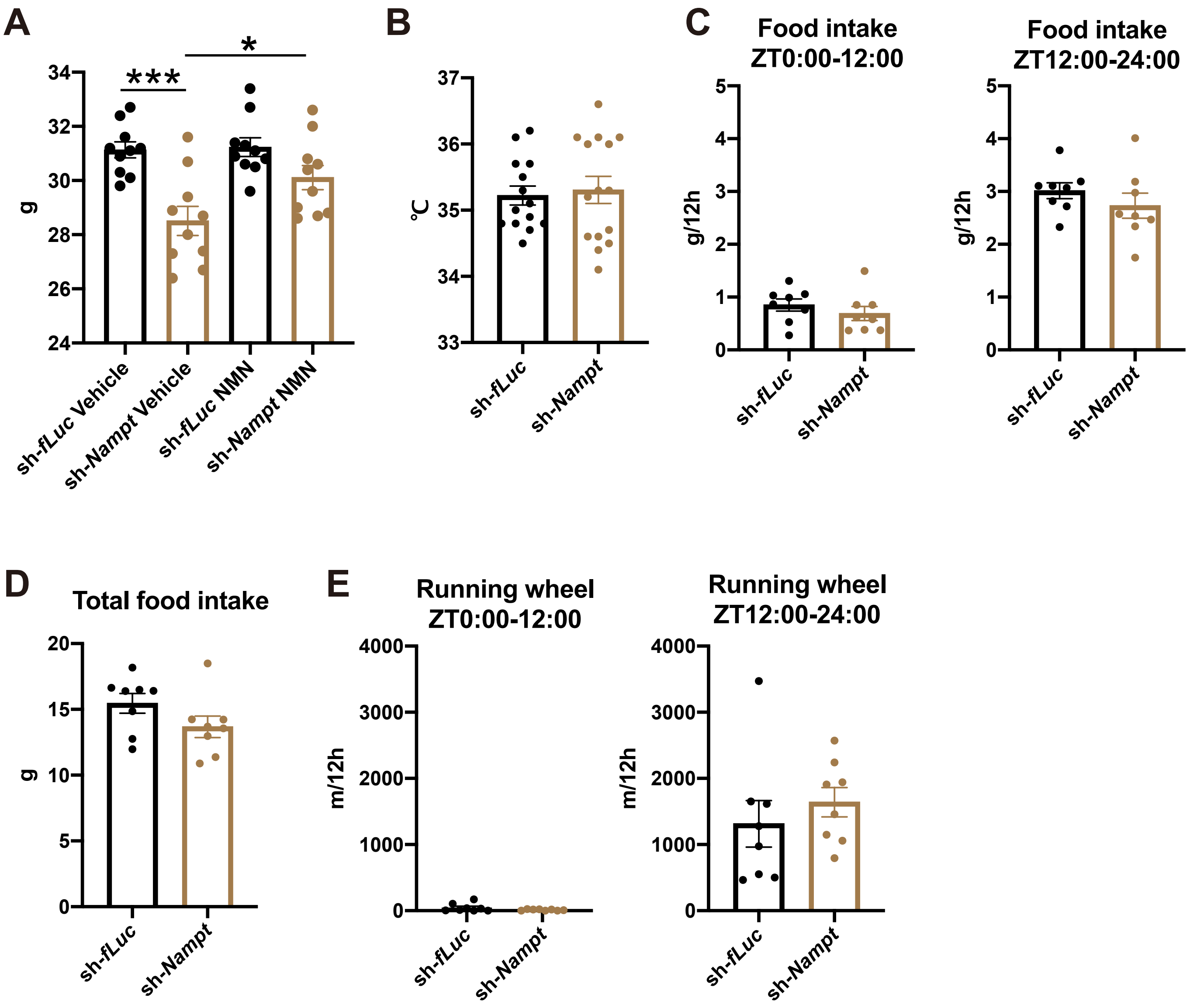

Figure S1 Related to Figure 1, *Nampt* in the LH regulates increases of body weight

(**A**) Effect of NMN on body weight in LH-specific *Nampt*-knockdown mice. n=10.

(**B**) Rectal temperature of LH-specific *Nampt*-knockdown mice. n=14-15.

(**C**) Average food intake during ZT0:00-12:00 (left) or ZT12:00-24:00 (right) in LH-specific *Nampt*-knockdown mice. n=8. (**D**) Accumulated food intake for 5 days in LH-specific *Nampt*-knockdown mice. n=8. (**E**) Average running distance by running wheel during ZT0:00-12:00 (left) or ZT12:00-24:00 (right) in LH-specific *Nampt*-knockdown mice. n=8. $^{*}P < 0.05$ and $^{***}P < 0.001$ by Tukey' s test for (**A**). Error bars indicate s.e.m.

Fig.S1

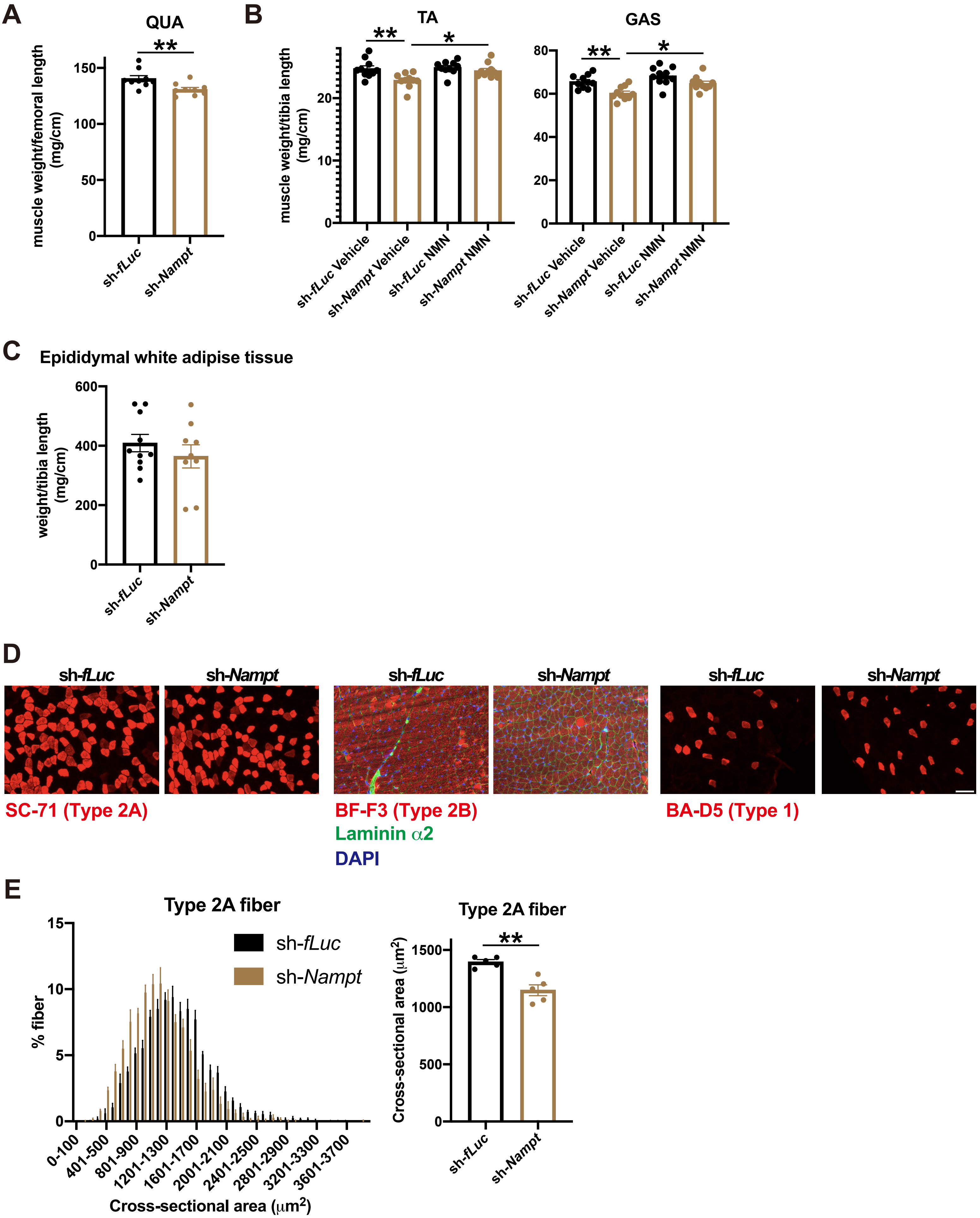
A
QUA
muscle weight/femoral length (mg/cm)
sh-fLuc
sh-Nampt
B
TA
GAS
muscle weight/tibia length (mg/cm)
sh-fLuc Vehicle
sh-Nampt Vehicle
sh-fLuc NMN
sh-Nampt NMN
C
Epididymal white adipise tissue
weight/tibia length (mg/cm)
D
sh-fLuc
sh-Nampt
SC-71 (Type 2A)
BF-F3 (Type 2B)
Laminin α2
DAPI
BA-D5 (Type 1)
E
Type 2A fiber
% fiber
Cross-sectional area (μm2)
Cross-sectional area (μm2)

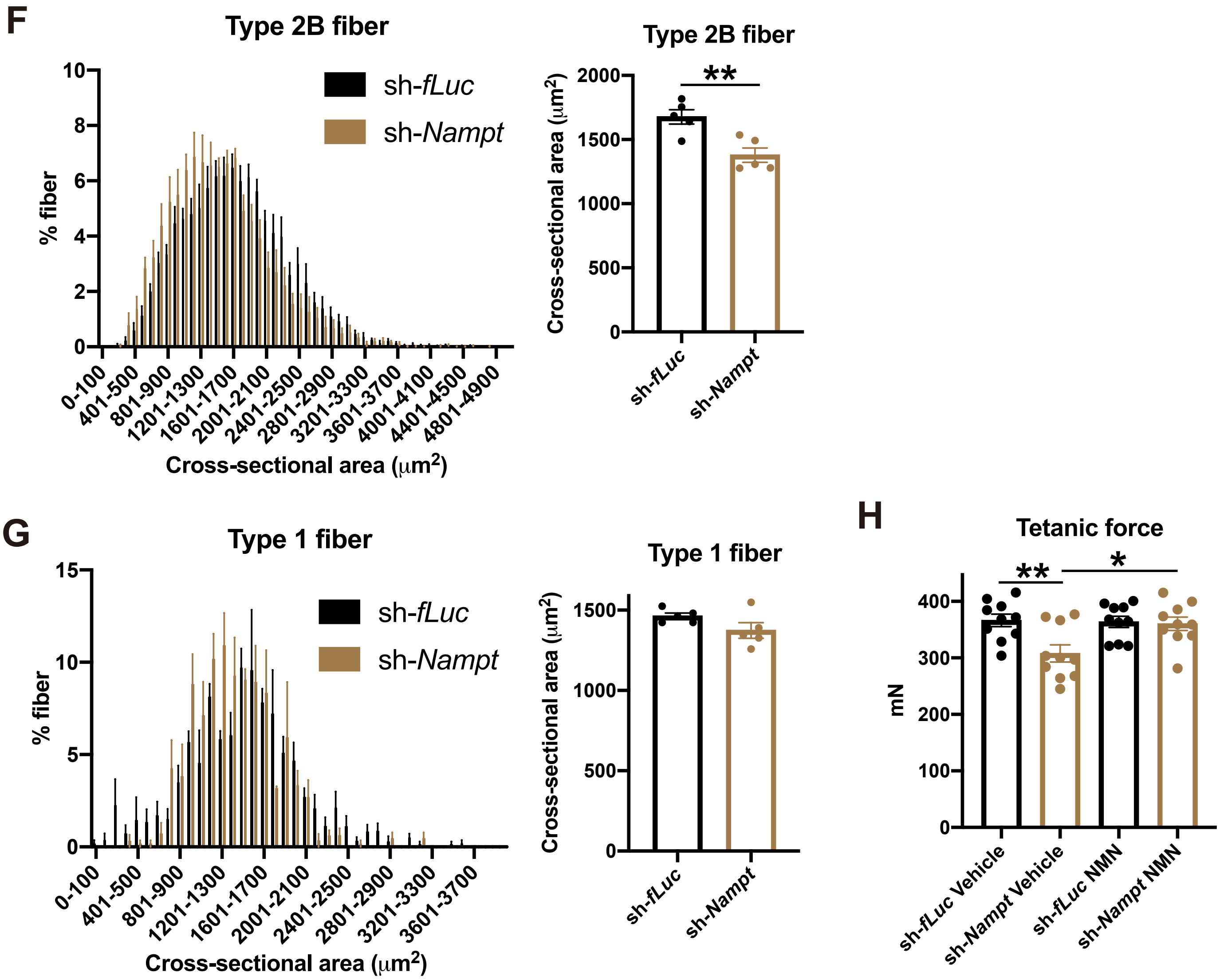

Figure S2 Related to Figure2, *Nampt* in the LH maintains muscle mass and force in skeletal muscle

(**A**) QUA muscle weights of LH-specific *Nampt*-knockdown mice normalized by femoral length. n=9. (B) Changes of TA and GAS muscle weights in LH-specific *Nampt*-knockdown mice with or without NMN administration. n=10. (**C**) Epididymal white adipose tissue weights of LH-specific *Nampt*-knockdown mice. n=9–10.

(**D**) Representative image for type 2A (left, red), type 2B (middle, red) and type 1 fibers (right, red). Type 2B fibers were co-stained with laminin α2 (middle, green) and DAPI (middle, blue). Bar: 100 μm. (**E-G**) Left: The cross-sectional areas of type2A fiber (**E**), type2B fiber (**F**) or type 1 fiber (**G**). Right: The average cross-sectional areas of type2A fiber (**E**), type2B fiber (**F**) or type 1 fiber (**G**). n=5. (**H**) In vivo tetanic force of LH-specific *Nampt*-knockdown mice with or without NMN administration. n=10. *$P < 0.05$ and **$P < 0.01$ by Student' s t-test for (A), (E) and (F) or by One-way ANOVA with Tukey' s test for (B) and (H). Error bars indicate s.e.m.

Fig.S2

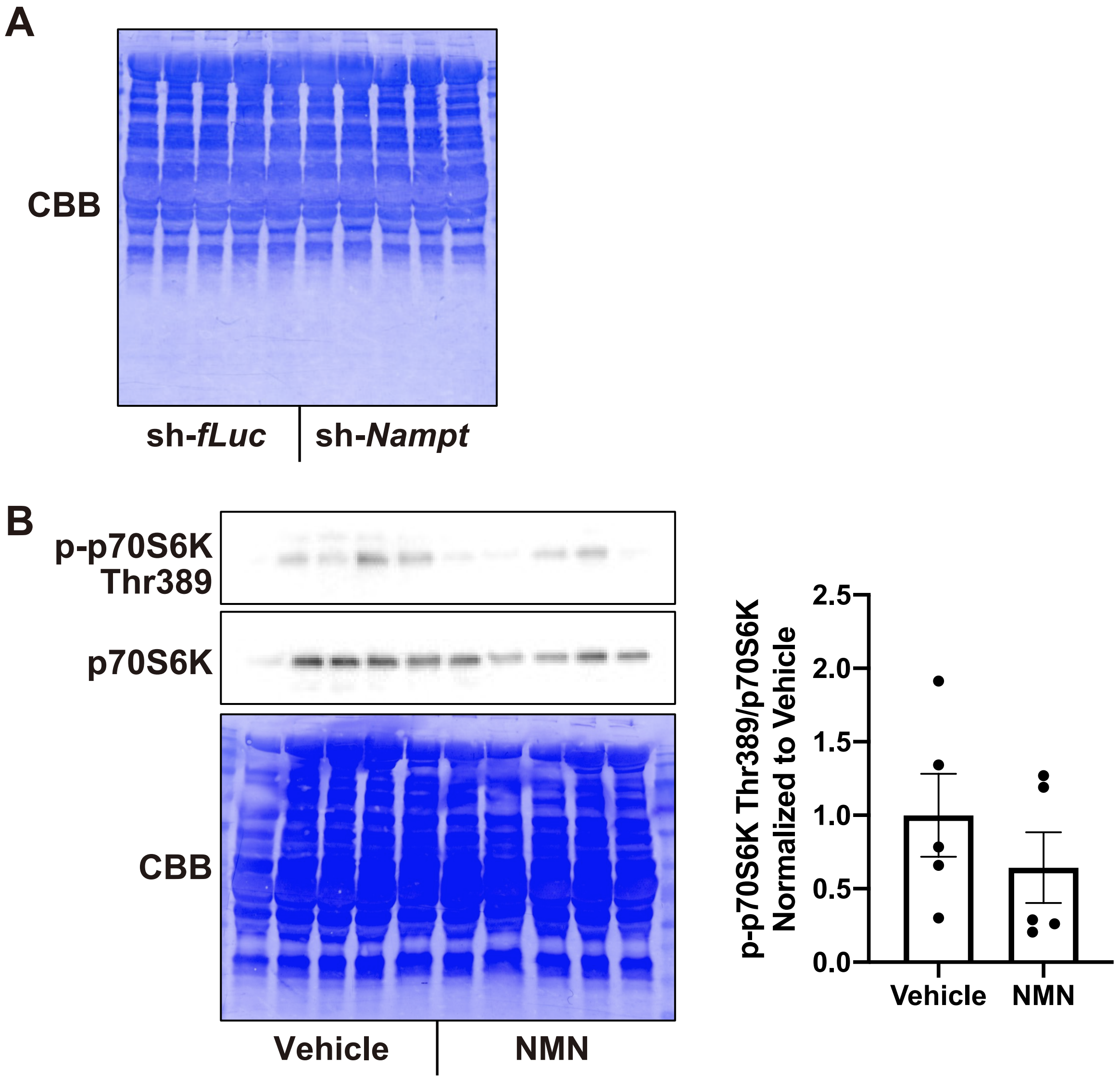

Figure S3 Related to Figure 3, *Nampt* in the LH regulates protein synthesis in skeletal muscle

(**A**) Representative CBB image of Figure 3A. (**B**) Left: Representative Western blot analysis showing phosphorylated and total p70S6K in TA muscle of NMN-injected mice.
Right: Quantitative analysis for the phosphorylation levels of p70S6K in TA muscle of NMN-injected mice. n=5.

Fig.S3

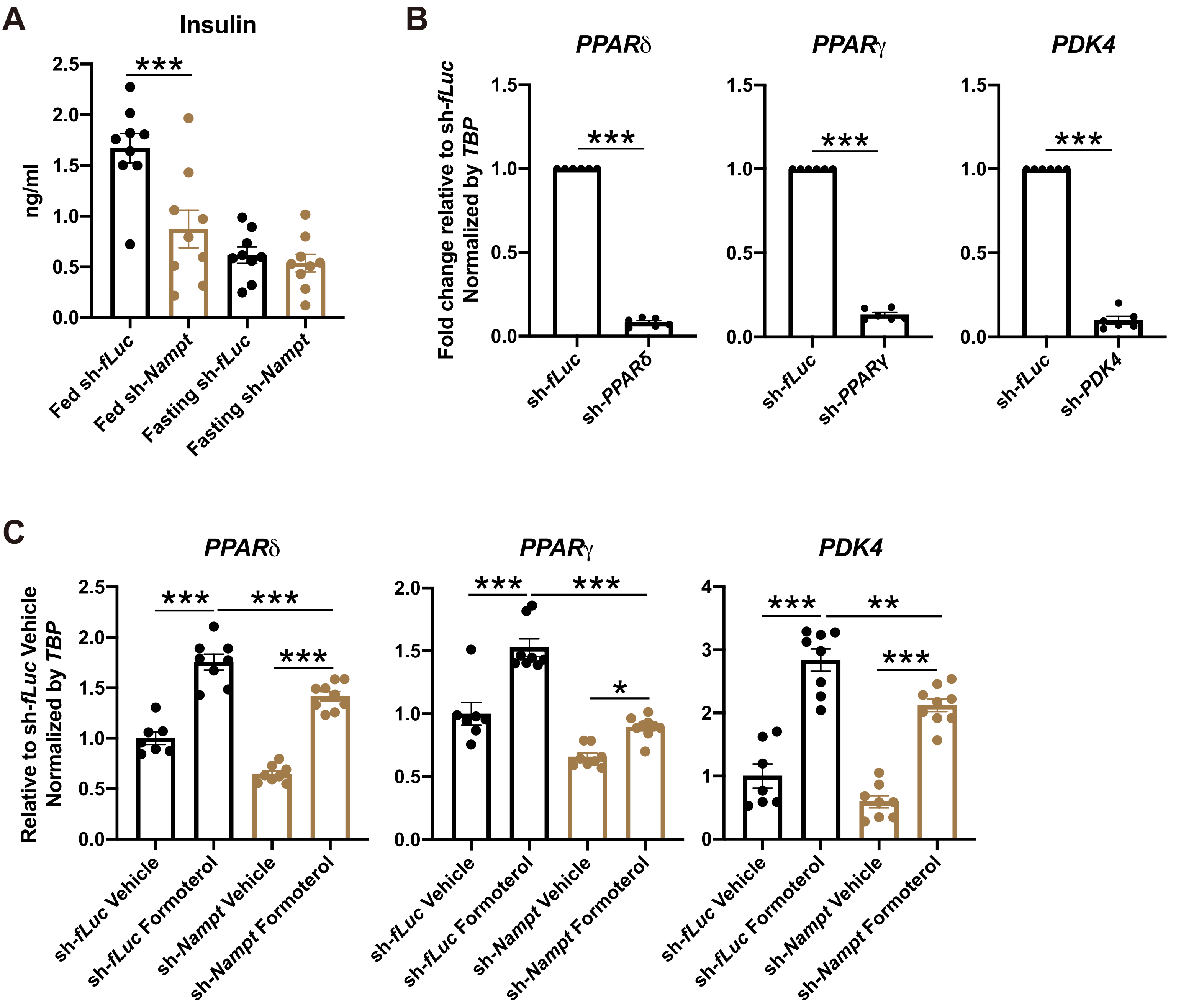

Figure S4 Related to Figure 4, *Nampt* in the LH regulates glycolysis in skeletal muscle

(**A**) Serum insulin levels at ZT 13:00 and after 24h after fasting in LH-specific *Nampt*-knockdown mice. n=9. (**B**) Knockdown efficiency of sh-*PPAR*δ, sh-*PPAR*γ or sh-*PDK4* in primary myotubes. n=6. (**C**) Expression of *PPAR*δ, *PPAR*γ and *PDK4* in TA muscle of formoterol-injected LH-specific *Nampt*-knockdown mice. n= 7-9. *P < 0.05, **P < 0.01 and ***P < 0.001 by Tukey’ s test for (**A**) and (**C**), or Student’ s t-test for (**B**). Error bars indicate s.e.m.

Fig.S4

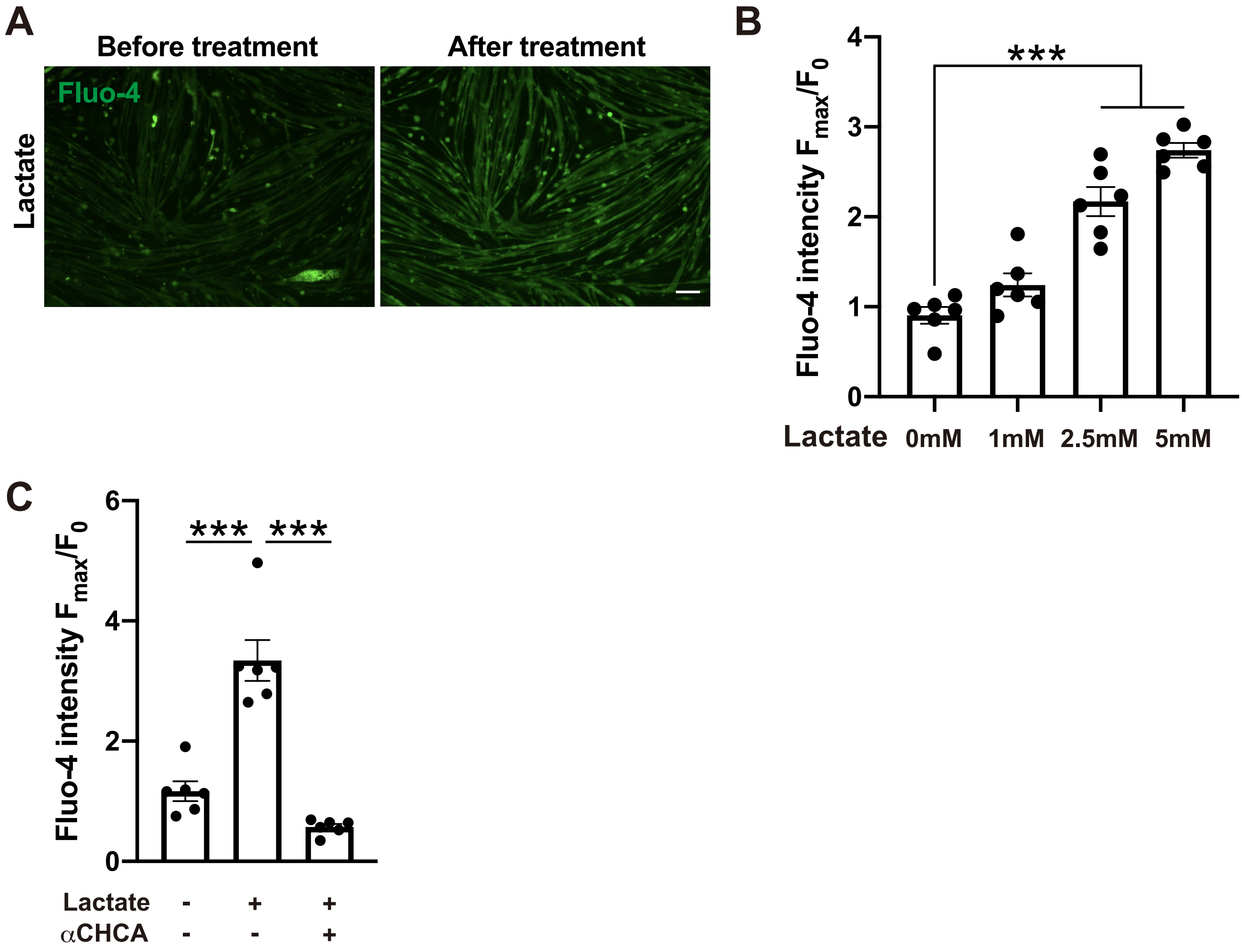

Figure S5 Related to Figure 5, Lactate induces increases in intracellular Ca^{2+} levels

(**A**) Representative fluorescent Fluo-4 images of primary myotubes before and after treatment with lactate. Bar: 100 μm. (**B**) Dose dependent changes of Fluo-4 intensity in lactate-treated C2C12 myotubes. n=6 (**C**) Lactate-induced increases of Fluo-4 intensity was inhibited by co-treatment with αCHCA. n=6. ***$P < 0.001$ by one-way ANOVA with Dunnett’ s test for (B), or one-way ANOVA with Tukey’ s test for (**C**).

Error bars indicate s.e.m.

Fig.S5

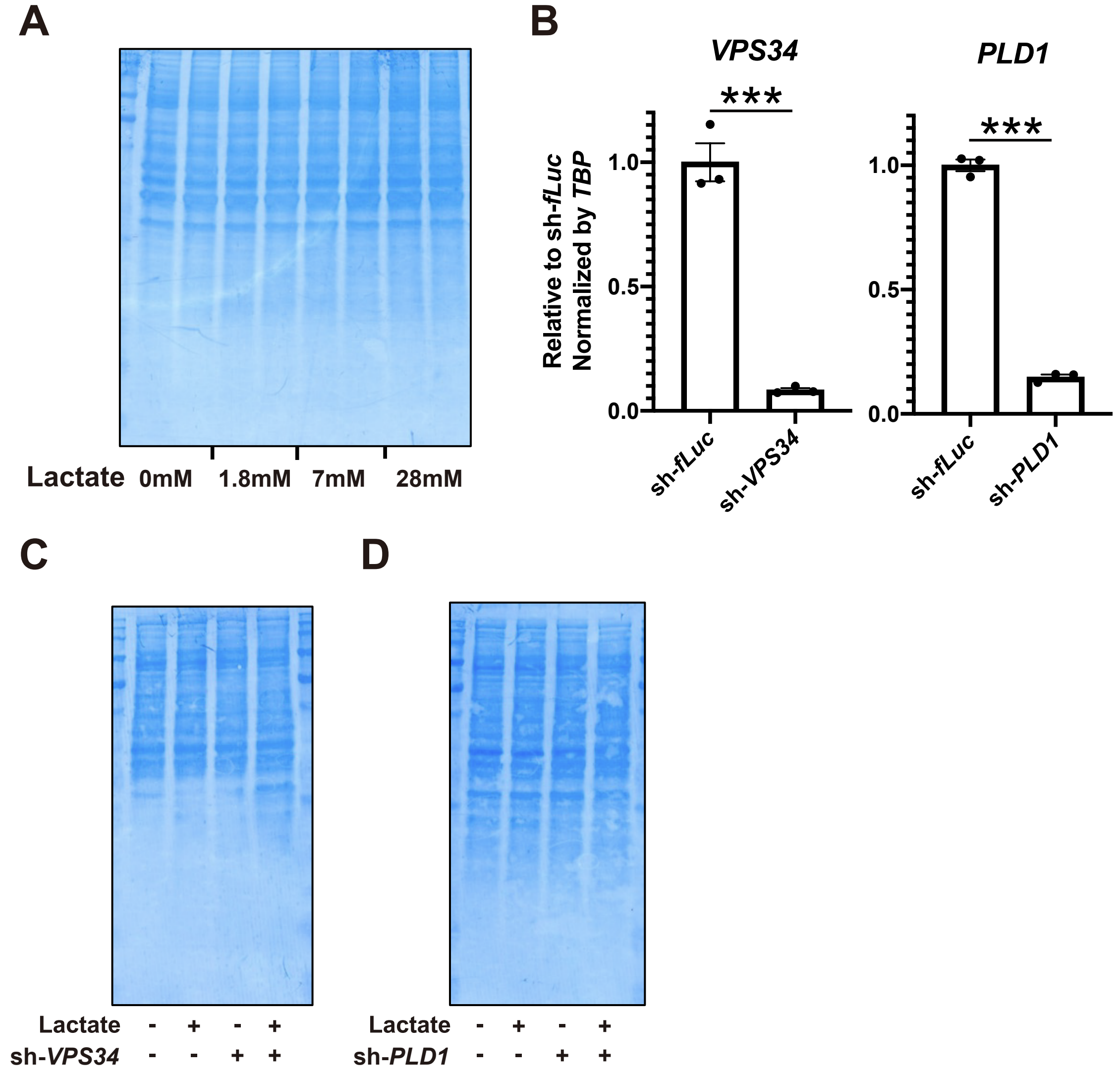

Figure S6 Related to Figure 6, Lactate induces activation of p70S6K-S6 axis through class III PI3K

(**A**) Representative CBB image for Figure 6A. (**B**) Knockdown efficiency of sh-*VPS34* or sh-*PLD1* in primary myotubes. n=3. (**C-D**) Representative CBB image for Figure 6C and 6D. ***P < 0.001 by Student’ s t-test. Error bars indicate s.e.m.

Fig.S6

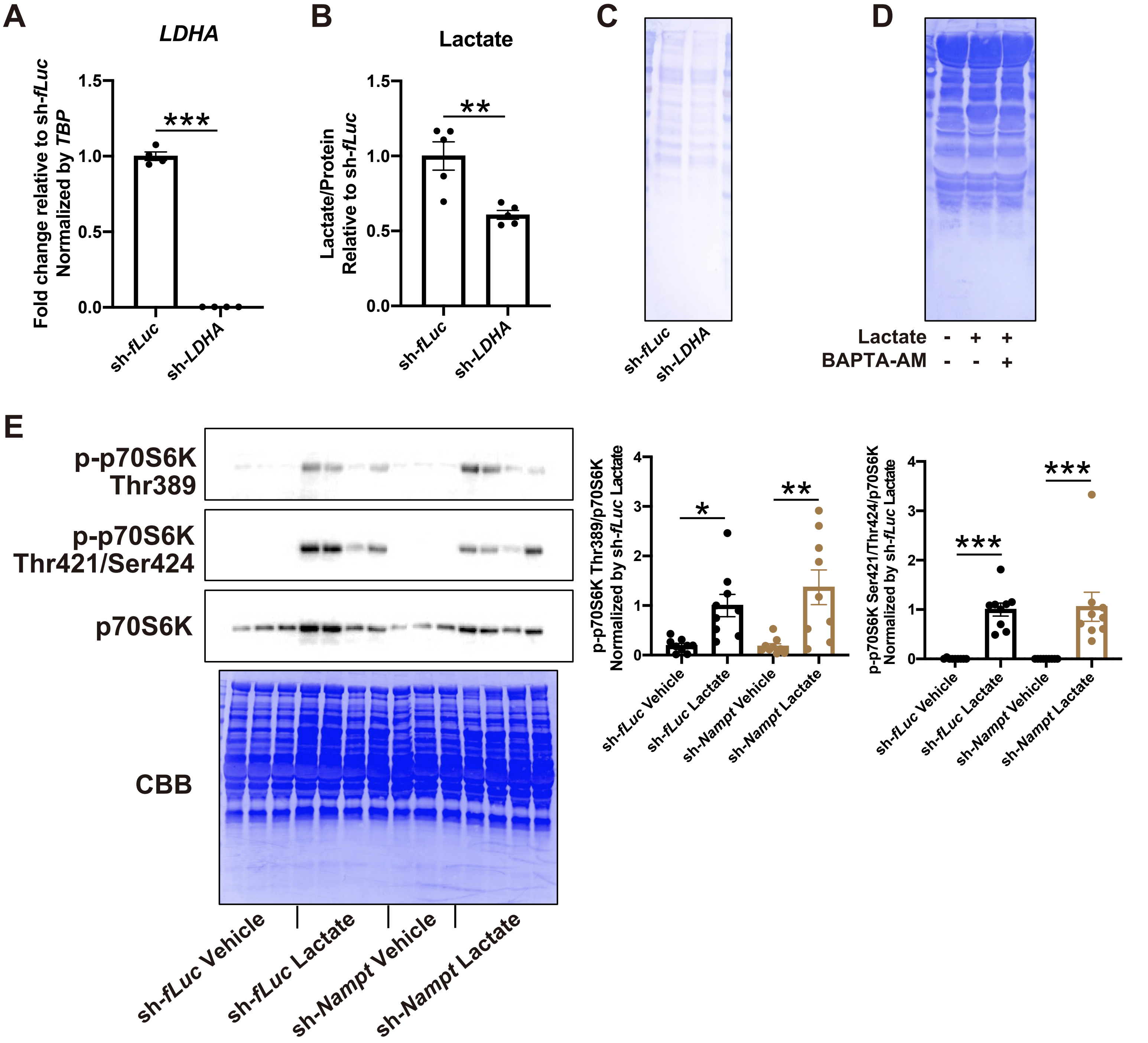

Figure S7 Related to Figure 7, Lactate-mediated Ca^{2+} signaling as an upstream regulator of p70S6K-S6 axis

(**A**) Knockdown efficiency of sh-*LDHA* in primary myotubes. n=4. (**B**) Lactate levels in *LDHA*-knockdown primary myotubes. n=5. (**C-D**) Representative CBB image for Figure 7A and 7B. (**E**) Left: Representative Western blot analysis showing phosphorylated and total p70S6K in lactate-injected TA muscle of LH-specific *Nampt*-knockdown mice.

Right: Quantitative analysis for the phosphorylation levels of p70S6K in lactate-injected TA muscle of LH-specific *Nampt*-knockdown mice. n=9.

*P < 0.05, **P < 0.01 and ***P < 0.001 by Student' s t-test for (A) and (B), or one-way ANOVA with Tukey' s test for (E). Error bars indicate s.e.m.

Fig.S7

Immunohistochemistry

Antibody	Source	Dilution
anti-GFP	NACALAI TESQUE, 04404-26	1:1,000
anti-Nampt	Thermo Fisher Scientific, A300-372A	1:600
Anti-type 1 fiber	Developmental Studies Hybridoma Bank, clone: BA-D5	1:300
anti-type 2A fiber	Developmental Studies Hybridoma Bank, clone: SC-71	1:200
anti-type 2B fiber	Developmental Studies Hybridoma Bank, clone: BF-F3	1:400
anti-laminin α2	Enzo Life Sciences, clone: 4H8-2	1:200
Alexa488 anti-Rat IgG	Thermo Fisher, A-11006	1:1,000
Alexa555 anti-Rabbit IgG	Thermo Fisher, A-21428	1:1,000
Alexa555 anti-Mouse IgG2b	ThermoFisher, A-21147	1:1,000
Alexa555 anti-Mouse IgG1	ThermoFisher, A-21127	1:1,000
Alexa555 anti-Mouse IgM	Thermo Fisher, A-21426	1:1,000

Western blot

Antibody	Source	Dilution
Anti-p-p70S6K (Thr389)	CST, #9205	1:400 for in vivo sample<br>1:2,000 for in vitro sample
anti-p-p70S6K (Thr421/Ser424)	CST, #9204	1:2,000 for in vivo sample<br>1:4,000 for in vitro sample
anti-p70S6K	CST, #2708	1:6,000,000 for in vivo sample<br>1:200,000 for in vitro sample
anti-p-S6 (Ser235/236)	CST, #4858	1:40,000 for in vivo sample<br>1:100,000 for in vitro sample
anti-S6	CST, #2217	1:20,000 for in vivo sample<br>10,000 for in vitro sample
anti-p-Akt (Ser473)	CST, #9271	1:2000 for in vivo sample<br>1:3,000 for in vitro sample
anti-Akt	CST, #9272	1:10,000
anti-p-FoxO1 (Ser256)	CST, #9461	1:1,200
anti-FoxO1	CST, #2880	1:4,000
anti-puromycin antibody	Millipore, MABE343	1:1,000
HRP-conjugated anti-rabbit IgG	GE, NA9340	1:2,000
EasyBlot HRP-conjugated goat anti-mouse IgG	GeneTex, GTX221667-01	1:2,000

Table.S1

Oligonucleotides

Nampt forward: 5′ -agaagttgcaggaggggatt-3′
Nampt reverse: 5′ -gctgctggaacagaatagcc-3′
PPARα forward: 5′ -agaagttgcaggaggggatt-3′
PPRAα reverse: 5′ -ttgaaggagctttgggaaga-3′
PPARδ forward: 5′ -tggagctcgatgacagtgac-3′
PPRAδ reverse: 5′ -tgtcctggatggcttctacc-3′
PPARγ forward: 5′ -gatggaagaccactcgcatt-3′
PPARγ reverse: 5′ -aaccattgggtcagctcttg-3′
PDK4 forward: 5′ -tgactcaaagacgggaaacc-3′
PDK4 reverse: 5′ -actggtcgcagagcatcttt-3′
MuRF1 forward: 5′ -atggagaacctggagaagcagc-3′
MuRF1 reverse: 5′ -tggaagatgtcgttggcacac-3′
atrogin-1 forward: 5′ -ccagcacacgacaacacttcag-3′
atrogin-1 reverse: 5′ -tatcccccgcagtttcaagc-3′
LDHA forward: 5′ -tggcagcctcttccttaaaa-3′
LDHA reverse: 5′ -gcggtgataatgaccagctt-3′
VPS34 forward: 5′ - gagacttcaggccttgcttg-3′
VPS34 reverse: 5′ - gggatgatgccccttatttt-3′
PLD1 forward: 5′ - agatgctatcgcccagagaa-3′
PLD1 reverse: 5′ - gtggaaatgtctccctcgaa-3′
TBP forward: 5′ -cagcctcagtacagcaatcaac-3′
TBP reverse: 5′ -taggggtcataggagtcattgg-3′

Table.S2
